# Phase Transformation Induced by High Pressure Torsion in the High-Entropy Alloy CrMnFeCoNi

**DOI:** 10.3390/ma15238407

**Published:** 2022-11-25

**Authors:** Robert Chulist, Aurimas Pukenas, Paul Chekhonin, Anton Hohenwarter, Reinhard Pippan, Norbert Schell, Werner Skrotzki

**Affiliations:** 1 Institute of Metallurgy and Materials Science, Polish Academy of Sciences, 30-059 Krakow, Poland; 2 Institute of Solid State and Materials Physics, Technische Universität Dresden, D-01062 Dresden, Germany; 3 Helmholtz-Zentrum Dresden-Rossendorf, D-01328 Dresden, Germany; 4 Chair of Materials Physics, Department of Materials Science, Montanuniversität Leoben, Jahnstraße 12, 8700 Leoben, Austria; 5 Erich Schmid Institute of Materials Science, Austrian Academy of Sciences, Jahnstraße 12, 8700 Leoben, Austria; 6 Institute of Materials Physics, Helmholtz-Zentrum Hereon, Max-Planck-Strasse 1, D-21502 Geesthacht, Germany

**Keywords:** high-entropy alloy, high pressure torsion, microstructure, texture, phase transformation, strength

## Abstract

The forward and reverse phase transformation from face-centered cubic (fcc) to hexagonal close-packed (hcp) in the equiatomic high-entropy alloy (HEA) CrMnFeCoNi has been investigated with diffraction of high-energy synchrotron radiation. The forward transformation has been induced by high pressure torsion at room and liquid nitrogen temperature by applying different hydrostatic pressures and large shear strains. The volume fraction of hcp phase has been determined by Rietveld analysis after pressure release and heating-up to room temperature as a function of hydrostatic pressure. It increases with pressure and decreasing temperature. Depending on temperature, a certain pressure is necessary to induce the phase transformation. In addition, the onset pressure depends on hydrostaticity; it is lowered by shear stresses. The reverse transformation evolves over a long period of time at ambient conditions due to the destabilization of the hcp phase. The effect of the phase transformation on the microstructure and texture development and corresponding microhardness of the HEA at room temperature is demonstrated. The phase transformation leads to an inhomogeneous microstructure, weakening of the shear texture, and a surprising hardness anomaly. Reasons for the hardness anomaly are discussed in detail.

## 1. Introduction

High-entropy alloys (HEAs) are single-phase, multi-element (≥5), solid solution alloys with near-equiatomic concentrations of the individual elements [1]. Among the reported HEAs with simple crystal structures, the most thoroughly studied alloy is the quinary equiatomic face-centered cubic (fcc) HEA CrMnFeCoNi [2] often referred to as Cantor alloy. This alloy is stable as a fcc single-phase solid solution at high temperatures above about 1073 K [3,4] but decomposes into several different metallic and intermetallic phases during annealing at intermediate temperatures [4,5,6,7]. In the fcc solid-solution state, the Cantor alloy exhibits certain noteworthy mechanical properties, including simultaneous strength and ductility increase with decreasing temperature leading to an outstanding fracture toughness at cryogenic temperatures [8]. At room temperature (RT) and below, above a certain stress (or strain), mechanical twinning contributes to deformation in addition to dislocation slip. Mechanical twinning is more pronounced at cryogenic temperatures and accounts for the excellent combination of strength and ductility at low temperatures.

The application of hydrostatic pressure at RT transforms the Cantor alloy to the hexagonal close-packed (hcp) structure (*ε*-martensite) [9]. The transformation is sluggish and occurs over a wide range of pressures. During depressurization, part of the hcp phase goes back to fcc. The reverse transformation is suppressed up to a high annealing temperature. Although the onset pressure of phase transformation reported varies widely, some trends are evident and may account for the large scatter. Increasing deviatoric stress, i.e., decreasing hydrostaticity, entails a decrease of the onset pressure, while decreasing grain size leads to an increase. These results are consistent with finite-temperature ab initio calculations, which show that the hcp structure is energetically favored at low temperatures [10,11,12]. The martensitic transformation in the Cantor alloy has been observed during high pressure torsion (HPT) at liquid nitrogen temperature (LNT, 77 K) and a pressure of 7.8 GPa [13] and 6 GPa [14]. However, despite large interest and ongoing research, the stability of the hcp phase remains far from being well-understood, especially for HPT samples deformed below RT and then investigated after heating-up to RT. 

In light of the above, the present work aims to show the effect of pressure during HPT at RT and LNT on the amount of martensitic transformation in the Cantor alloy and its effect on microstructure and texture formation and in particular on microhardness. Additionally, the stability of the hcp phase introduced by HPT at LNT as a function of long-time storage (over three years) at ambient conditions (RT, atmospheric pressure, air) is studied. 

## 2. Experimental

The CrMnFeCoNi HEA was synthesized from high-purity elements (>99.9 wt.%) by arc melting and drop casting under pure argon atmosphere into cylindrical molds (diameter: 25.4 mm, length: 127 mm). The drop-cast ingots were homogenized for 48 h at 1200 °C in evacuated quartz ampules. Discs with a diameter of 6 or 8 mm and an initial thickness *t*_i_ ≈ 0.8 mm were cut from the cast and homogenized ingots and deformed by HPT [15]. During HPT the shear strain *γ* along the radius *r* is approximately given by *γ = 2πrn/<t>*, where *n* is the number of rotations (5 or 10) and *<t>* = (*t*_i_ + *t*_f_)/2 with *t*_f_ = final thickness. With this approximation, the maximum error in shear strain is less than 15%. HPT under a quasi-hydrostatic nominal pressure of 4 to 10 GPa was conducted at RT and LNT at a nominal speed of 0.2 rotations/min, giving a maximum shear strain rate of about 10^−1^ s^−1^ at the outer radius; details of HPT at LNT are given in [16]. The initial grain size was several hundred micrometers, while the saturation microstructure after HPT at RT was estimated by transmission electron microscopy to consist of crystallites (grains) about 50 nm in size [4]. The crystallite size estimated by X-ray diffraction line-profile-analysis is approximately 24 nm for samples HPT deformed at RT and LNT [13]. During HPT at RT, the alloy does not decompose as confirmed by 3D atom probe tomography [4].

Texture measurements were performed by diffraction of synchrotron radiation at the high energy X-ray beamline HEMS P07B at PETRA III (DESY, Hamburg, Germany) [17], wavelength λ = 0.01423 nm, beam size (0.7 × 0.7) mm^2^, sample detector distance 1320 mm, and Mar345 flat panel detector with a pixel size of 150 μm. The sample was a pin of about (1 × 1 × 0.8) mm^3^ taken along the radial direction of the HPT disc. It was transmitted by the synchrotron beam with size given above and continuously 180° rotated in 36 steps of 5° with its long axis around the ω-axis. Details of torsion texture measurements with high-energy synchrotron radiation are given in [18]. The orientation distribution function (ODF) was calculated from the measured (200), (222) pole figures (PFs) (fcc phase) and (100) (101) and (102) (hcp phase) using LaboTex V3 software [19] and applying monoclinic sample symmetry (In this paper, both the Miller and Miller–Bravais notation is used). Measurements performed in transmission mode on bulk samples allowed to register complete PFs in a single experiment. The Euler angles given are in the Bunge notation [20] with crystal and sample reference systems defined as x || shear direction (SD), y || transverse direction (TD) and z || shear plane normal (SPN) (fcc: x, y, z = <100>, hcp: x = [110], y = [100], z = [001]). The textures of the fcc phase are represented by *φ*_2_ = 45° ODF-sections, those of the hcp phase by *φ*_2_ = 0° and 30° ODF-sections. These ODF-sections for both phases contain all major shear components.

In order to avoid the effect of texture on phase analysis, the samples were rotated 180° in one step around the pin long axis (ω-axis) perpendicular to the incident beam. The intensity was integrated over the whole Debye-Scherrer rings and the resulting patterns of the parent and martensitic phase were analyzed with the Rietveld refinement method using HighScore Plus software and the so-called continuous mode for synchrotron diffraction [21,22]. Such a procedure practically reduces the effect of crystallographic texture allowing very precise calculations of the phase volume fraction. The parameters used ensured good counting statistics with a typical value of goodness of fit parameter lower than 2 for all Rietveld quantifications. The mean Rietveld error of the phase volume fractions is 0.4%. 

A FEI Quanta 3D scanning electron microscope (FEI Company, Hillsboro, Oregon USA) equipped with an electron backscatter diffraction (EBSD) camera was used to characterize the microstructure and texture evolution of the HPT deformed samples. The acceleration voltage used was 12 kV. The pattern acquisition and indexing were done using the TSL OIM Analysis 7 software. The mappings were carried out in the beam-scanning mode with step sizes ranging between 40 and 60 nm. 

Vickers micro-hardness measurements were conducted with a microhardness tester (Buehler Micromet 5104, Buehler Ltd., Düsseldorf, Germany) at a load of 1000 gf and dwell times of 15 s. Measurements were taken on the disc-shaped samples along four orthogonal lines from the center to the edge, i.e., as a function of shear strain. 

## 3. Results

### 3.1. Phase Transformation

**Figure 1:**
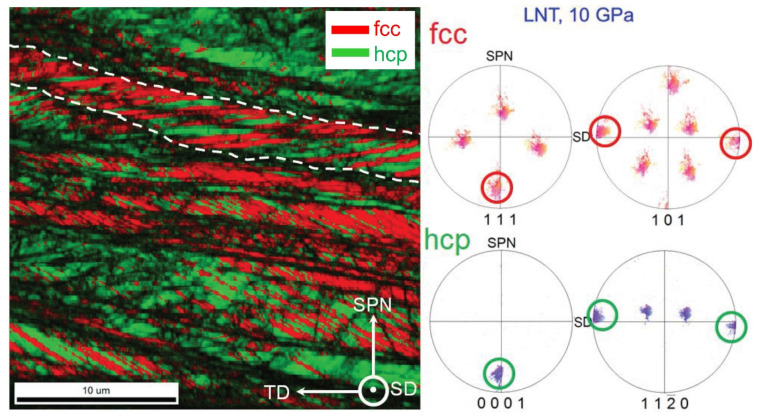
Distribution of fcc and hcp phase in a sample HPT-deformed at LNT and 10 GPa demonstrated by EBSD. The area shown represents a weakly deformed region. The pole figures of the encircled area demonstrate the orientation relationship of the fcc/hcp lamellae.

**Figure 2:**
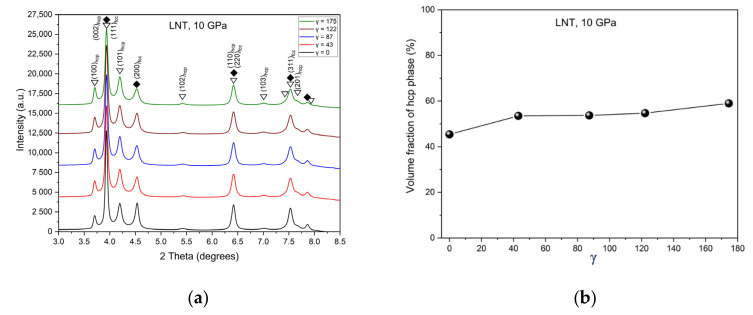
(**a**) X-ray diffractograms after HPT at LNT and 10 GPa for different shear strains *γ* clearly demonstrating the phase transformation. (**b**) Volume fraction of hcp phase as a function of shear strain.

The phase transformation from fcc to hcp during HPT was observed at RT and LNT with the onset pressure decreasing with decreasing temperature. Figure 1 shows an EBSD map of the phase distribution in a weakly deformed area after HPT at LNT and a pressure of 10 GPa. The transformation occurs in lamellar form along the {111} planes of the fcc parent phase: trace of the fcc/hcp lamellae in the encircled area is normal to the encircled [111] and [0001] direction in the (111)_fcc_ and (0001)_hcp_ PFs. The orientation relationship is shown by comparing the encircled maxima in the corresponding PFs: (111)_fcc_||(0001)_hcp_ and [101]fcc||[11 $\bar{2}0$] _hcp_. The phase transformation is rather heterogeneous, leading to an inhomogeneous microstructure which after severe plastic deformation (SPD) finally changes to a grain refined two-phase nanostructure. Figure 2a shows the diffractograms at different shear strains *γ* displaying the peaks of the hcp phase. It is interesting that only a small shear strain is necessary to transform the material to a high amount. In general, further straining increases the volume fraction only slightly (Figure 2b). 

**Figure 3:**
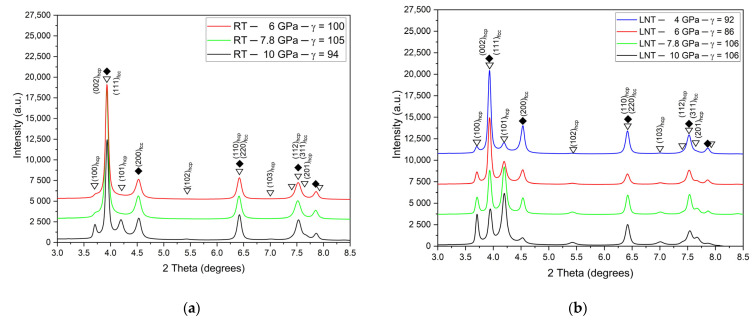
X-ray diffractograms after HPT under different pressures at RT (**a**) and LNT (**b**).

**Figure 4:**
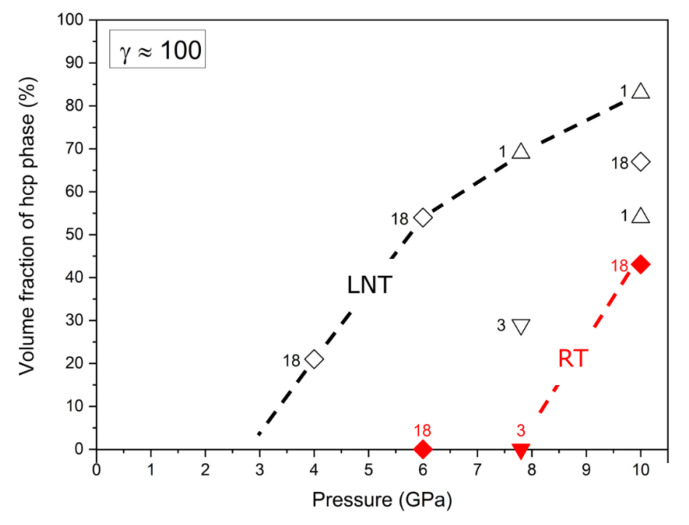
Volume fraction of martensitic phase versus pressure at a shear strain *γ* ≈ 100. The symbols represent different series of HPT samples, for which the phase composition was measured after the number of months indicated.

**Figure 5:**
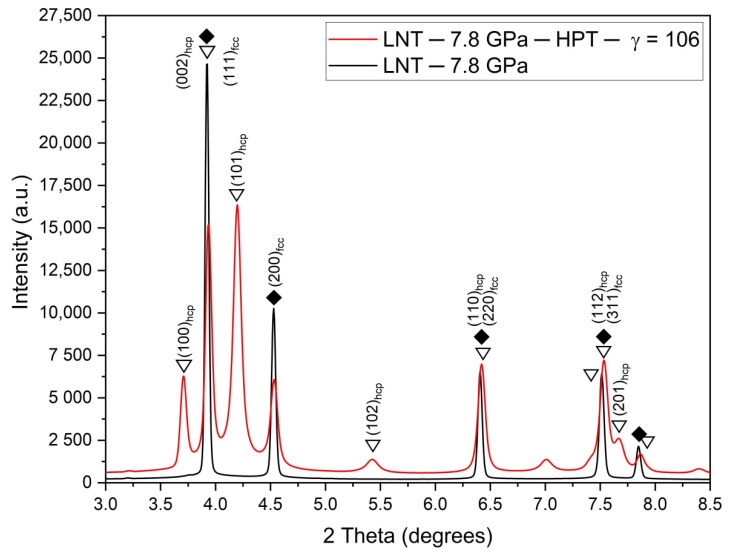
X-ray diffractograms after HPT at LNT clearly showing the necessity of shearing for inducing the phase transformation up to pressures of 7.8 GPa.

Figure 3a,b show the diffractograms after HPT under different pressures at RT and LNT, respectively, at a shear strain *γ* ≈ 100. While at LNT the phase transformation occurs at all pressures applied, at RT it is only present at the highest pressure of 10 GPa. The small (100)_hcp_ hump of the samples HPT deformed at RT and 6 and 7.8 GPa may be due to stacking faults in the fcc structure resulting from the dissociation of 1/2<110> dislocations. These stacking faults represent extended hcp layers of atomic thickness. The thin layers lead to the absence of the (101)_hcp_ peak due to the infinite broadening of peaks with (00l)_hcp_ components. The volume fraction of the martensitic phase increases with pressure with the onset pressure decreasing with decreasing temperature from about 8 GPa at RT to about 3 GPa at LNT (Figure 4). Because of the scatter of data and reverse transformation during storage at ambient conditions (s. Figure 11 below), exact onset pressures cannot be given. Figure 5 shows that even at LNT and a pressure of 7.8 GPa, additional shearing is necessary to induce the martensitic transformation.

### 3.2. Texture Formation

**Figure 6:**
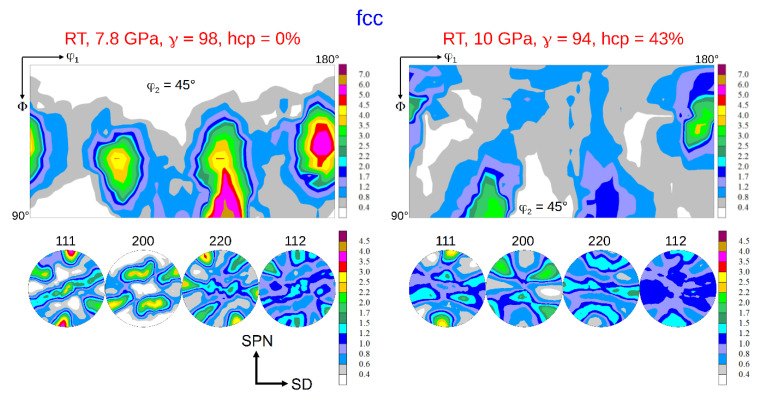
Texture of the fcc parent phase after HPT at RT and pressures of 7.8 GPa (no hcp phase) and 10 GPa (43 vol.% hcp phase) at a shear strain *γ* ≈ 100. (for key figure and designation of the fcc shear texture components see Figure 7).

**Figure 7:**
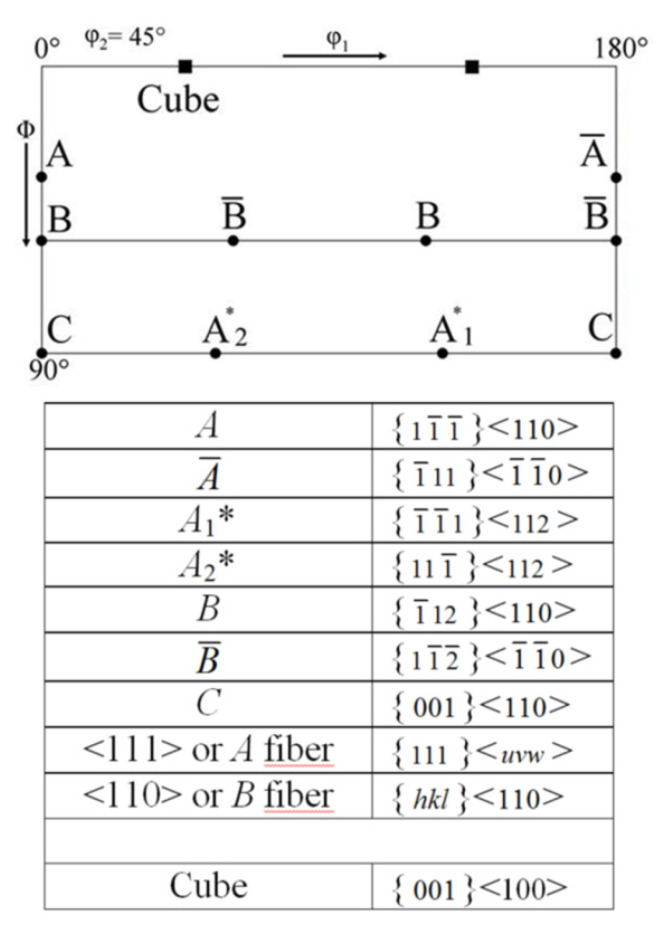
Key figure of shear texture components of HPT deformed fcc phase and their crystallographic description with regard to shear plane {hkl} and shear direction <uvw>. The cube component is also given for the texture analysis of Figure 12 below.

The texture of the fcc phase developing during HPT at RT is a typical shear texture of fcc metals with medium/low stacking fault energy (SFE) [13,23,24]. The dominant texture components are B resp. $\bar{B\;}$ and $A_{1}^{*}$ resp. $A_{2}^{*}$; minor components are A resp. $\bar{A\;}$ (Figure 6 left). The set of components $A_{1}^{*}/A_{2}^{*}$ is centro-symmetric or “self-symmetric” with respect to TD (twofold symmetry axis of torsion), while the sets A/$\bar{A\;}$ and B /$\bar{B\;}$ are “twin-symmetric”. For key figure and designation of the fcc shear texture components, see Figure 7. The texture strength decreases with increasing volume fraction of the hcp phase and the relative contributions of the texture components change (Figure 6 right). For HPT at LNT, the randomization is even stronger (s. Figures 9 and 10 below).

**Figure 8:**
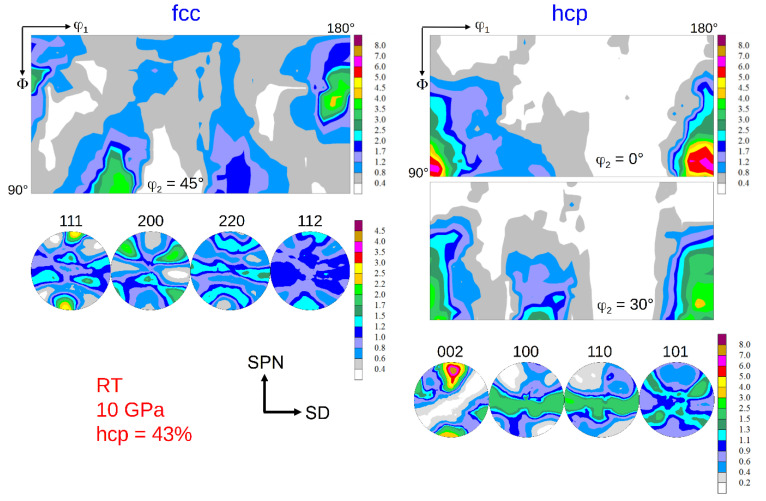
Texture of the fcc and hcp phase after HPT at RT at 10 GPa pressure and a shear strain *γ* = 94. The hcp volume fraction is 43%. (for key figures and designation of the fcc and hcp shear texture components see Figure 7 and Figure 13 below, respectively).

**Figure 9:**
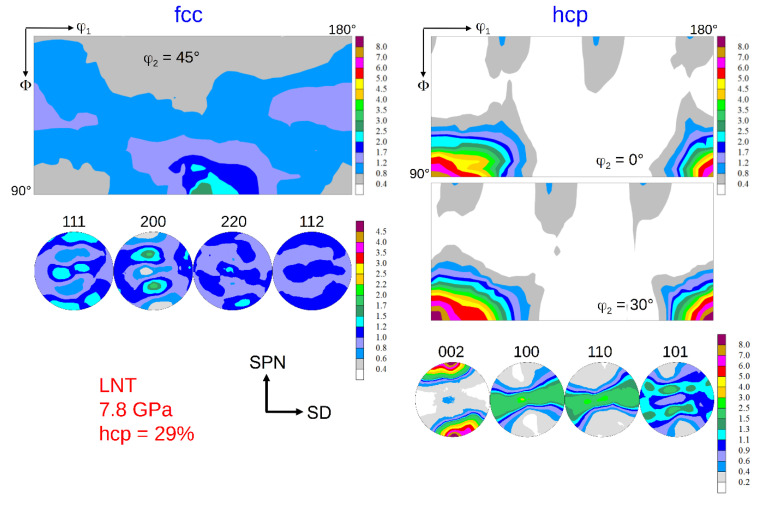
Texture of the fcc and hcp phase after HPT at LNT at 7.8 GPa pressure and a shear strain *γ* = 98. The hcp volume fraction is 29%. (for key figures and designation of the fcc and hcp shear texture components see Figure 7 and Figure 13 below, respectively).

**Figure 10:**
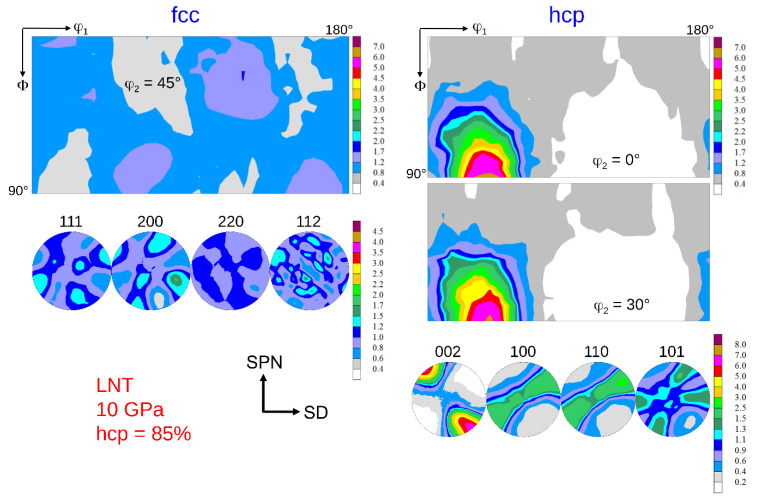
Texture of the fcc and hcp phase after HPT at LNT at 10 GPa pressure and a shear strain *γ* = 106 after 1 month storage at RT. The hcp volume fraction is 85%. (for key figures and designation of the fcc and hcp shear texture components see Figure 7 and Figure 13 below, respectively).

The texture of the hcp phase depends on the volume fraction transformed during HPT. For volume fractions smaller than about 50%, the texture is related to the texture of the parent fcc phase with the *c*-axis fibre parallel to the *A*-fibre (Figure 8 and Figure 9), i.e., (0001)_hcp_||(111)_fcc_ (compare Figure 7 and Figure 8 with Figure 13 below). This parallelism is easily seen by comparing the PFs (002)_hcp_ = (0001) and (100)_hcp_ = (10$\bar{1}0$) or (110)_hcp_ = (11$\bar{2}0$) with (111)_fcc_ and (110)_fcc_, respectively. For volume fractions larger than approximately 50%, the hcp phase dominates the deformation. Now, the *c*-axis fibre is rotated anticlockwise about TD by approximately 30° with respect to the SPN (Figure 10).

**Figure 11:**
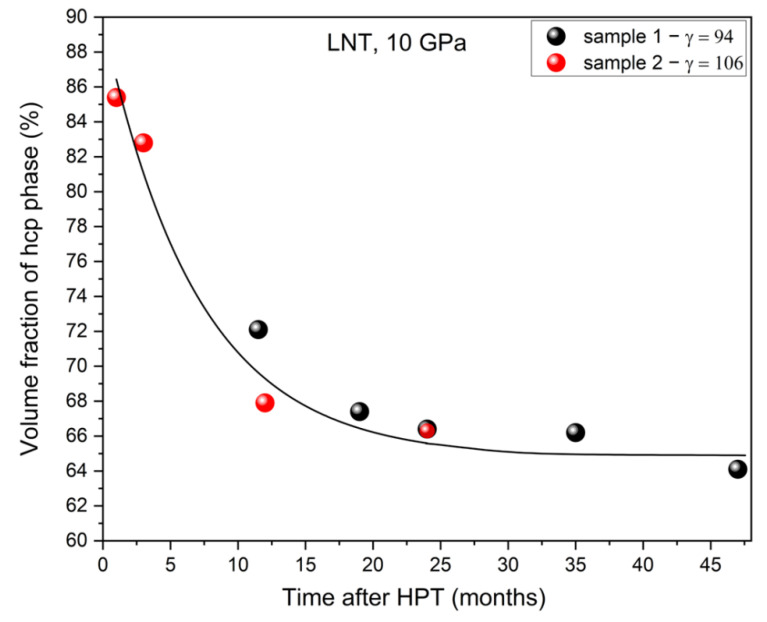
Reverse phase transformation of hcp to fcc during storage at ambient conditions investigated for two samples HPT-deformed at LNT.

Storage of the Cantor alloy HPT-deformed at LNT at ambient conditions results in a reverse transformation of hcp to fcc. This has been shown for two samples deformed at LNT and 10 GPa to a shear strain *γ* ≈ 100 (Figure 11). The volume fraction of hcp phase decreases with time from 85% to 64% after three years, i.e., by 25%, and then stays constant. 

**Figure 12:**
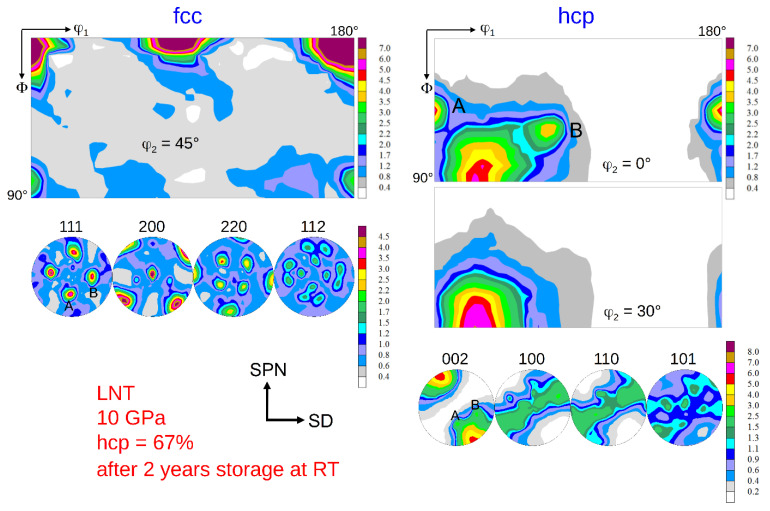
Texture of the fcc and hcp phase after HPT at LNT at 10 GPa pressure and a shear strain *γ* = 94 after 2 years storage at ambient conditions. The remaining hcp volume fraction is 67%. The two hcp recrystallization and/or grain growth components are marked *A* and *B* in the ODF and the (002)_hcp_ PF. The related components in the fcc phase are also marked in the (111)_fcc_ PF. (for key figures and designation of the fcc and hcp shear texture components see Figure 7 and Figure 13, respectively).

**Figure 13:**
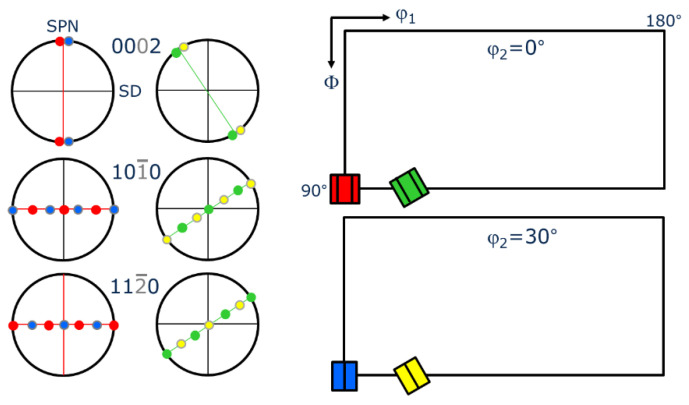
Key figure of shear texture components of HPT deformed hcp phase (**right**) and their crystallographic description with regard to shear plane and shear direction (**left**).

Besides reverse transformation, self-annealing during long-term storage at RT leads to recrystallization changing the texture, too (Figure 12). A new component of the fcc phase appearing in the sample stored over two years at ambient conditions (Figure 10 left) is a 45° TD-rotated (oblique) cube component. This texture differs considerably from the initially almost random texture (after 1 month storage). In the hcp phase in the φ_2_ = 0° ODF section, two new components *A* and *B* emerge at Euler angles (0, 45, 0) and (60, 70, 0), respectively. Their basal planes are related to the {111} planes of the fcc oblique cube recrystallization component; see *A* and *B* in the (111)_fcc_ PF.

### 3.3. Strength Development

**Figure 14:**
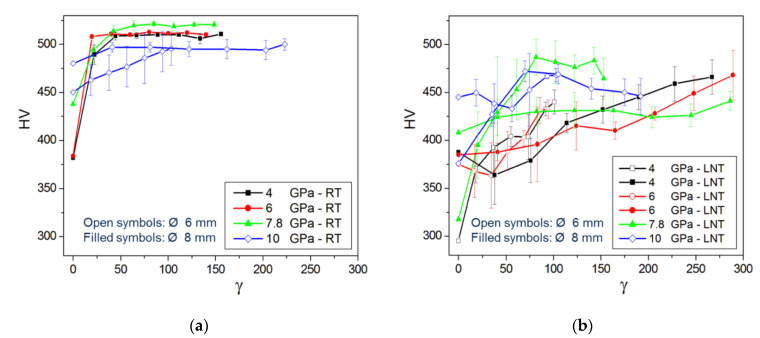
Microhardness measured at RT of samples HPT-deformed at RT (**a**) and LNT (**b**) versus shear strain *γ*.

**Figure 15:**
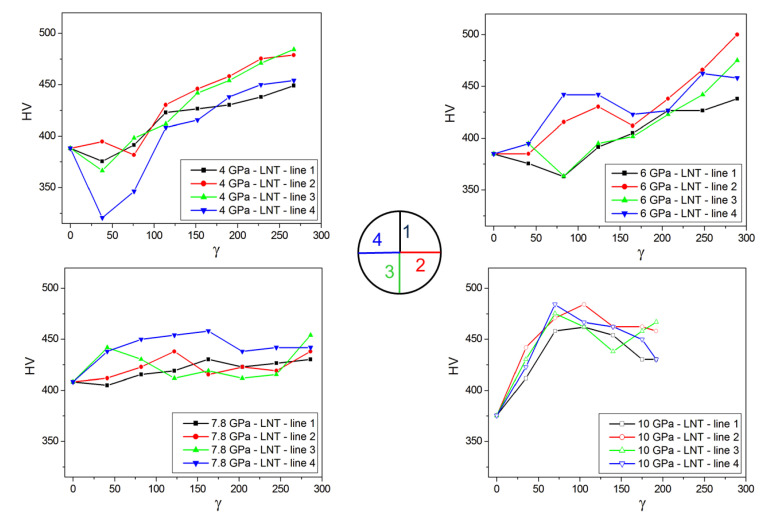
Microhardness measured along 4 radial directions (lines) at RT of samples HPT-deformed under different pressures at LNT versus shear strain *γ*.

**Figure 16:**
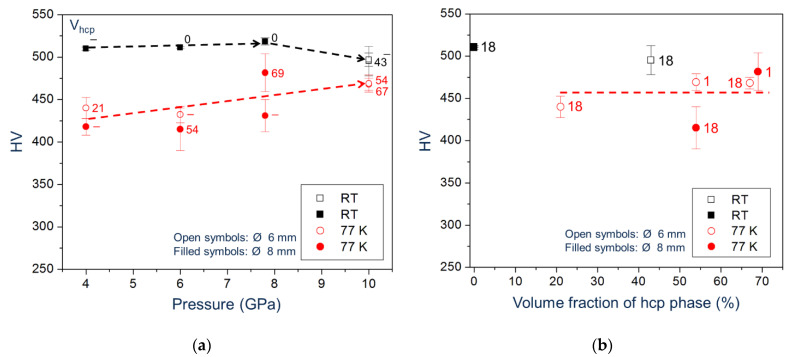
Microhardness measured at RT versus pressure (**a**) and volume fraction of hcp phase (**b**) of samples HPT-deformed at RT and LNT (*γ* ≈ 100). The volume fraction of hcp phase (**a**) and time of phase analysis after HPT (in months) (**b**) is given.

Figure 14a,b show the Vickers hardness as a function of shear strain measured at RT for HPT samples deformed at RT and LNT, respectively. The RT-microhardness increases with shear strain and saturates at a certain strain level depending on pressure and HPT temperature. While the saturation microhardness slightly increases with pressure up to 7.8 GPa, it decreases at 10 GPa because of the phase transformation. In addition, the microhardness values show a larger scatter. Similarly, in the case of HPT at LNT, there is a large scatter of the microhardness resulting from the large spread within the HPT disk as shown by the measurements along different radial directions (Figure 15). The scatter is more pronounced at low shear strains and becomes smaller with increasing pressure. Despite the large scatter, it is evident that a kind of microhardness saturation takes place at much higher strains with the transition strain decreasing with pressure. Moreover, there is a clear microhardness anomaly: (490–520) HV for RT HPT, in contrast to (420–480) HV for LNT HPT (Figure 16). There seems to be no correlation between microhardness and the amount hcp phase, but only with pressure. The values for RT and LNT HPT-deformed samples seem to approach each other for pressures higher than 10 GPa. 

## 4. Discussion

### 4.1. Phase Transformation Process

Hydrostatic pressure applied to the Cantor alloy stabilizes the hcp structure [9]. In order to transform the fcc phase to hcp, a certain onset pressure is required depending on hydrostaticity and grain size. On the one hand, the use of different media for pressurization reduces the onset pressure at RT from 22.1 GPa (helium) to 6.9 (silicone oil) and (2.2–6.6) GPa (amorphous boron), i.e., with decreasing hydrostaticity [25,26]. The same effect was also observed for iron [27]. On the other hand, for a given medium (silicone oil), decreasing the grain size from 5 μm to about 0.01 μm increases the onset pressure from 6.9 GPa to 12.3 GPa [26]. However, the origin of this effect is not well understood. The fcc to hcp martensitic transformation occurs through slip of 1/6 <112> Shockley partial dislocations on every second {111}_fcc_ plane [28], giving an orientation relationship (111)_fcc_||(0001)_hcp_ and [110]fcc||[11$\bar{2}0$] _hcp_. The nucleation of the phase transformation by tearing apart of dissociated 1/2<110> dislocations is easier the lower the SFE. After nucleation the phase transformation under decreasing load continues at almost the speed of sound. The participating partial dislocations possess such a large kinetic energy, such that in a pole mechanism they are able to pass each other dynamically at closer range than would be possible under quasi-static conditions [29]. Thus, shear stresses support the nucleation process as well as the motion of partial dislocations along (111)_fcc_/(0001)_hcp_ interphase boundaries. HPT at RT did not lead to the transformation below about 8 GPa. The reasons for this may be an insufficient difference in the Gibbs free energy of the phases or the fast grain refinement of both phases during SPD into a globular nanocrystalline structure suppressing the transformation similar to deformation twinning [30]. However, lowering the HPT temperature to 77 K, i.e., lowering the SFE [10,12], favours the transformation at the pressures applied. 

According to finite-temperature ab initio calculations in the Cantor alloy the hcp structure is energetically favored at low temperatures [10,11,12]. However, in situ X-ray diffraction during cooling down to 3 K did not reveal any phase transformation from fcc to hcp [31]. The hcp phase was also absent after large tensile deformation (true strain of 35%) at 4 K as was proven by highly sensitive X-ray diffraction with high-energy synchrotron radiation at RT (unpublished). On the other hand, the hcp phase produced by hydrostatic compression at RT is quite stable during decompression and heating up above RT [25], but nevertheless partly transforms back to fcc. Similarly, the results presented here show that there is a reverse transformation of the hcp phase produced by HPT at LNT during long-term storage under ambient conditions. These results strongly suggest that the fcc structure is the thermodynamically stable structure at low temperatures, contrary to theory. However, the hcp structure becomes stable under hydrostatic pressure. The sluggishness of the forward and reverse transformation may be due to the difficult movement of partial dislocations in the concentrated alloy producing the displacive transformation. For further discussion of the discrepancy between experiment and theory on the phase stability the reader is referred to [32,33].

### 4.2. Effect of Phase Transformation on Microstructure Development

Phase transformation via hcp nanolamellae leads to an inhomogeneous microstructure because transformation sets-in in favourably oriented grains, changing their slip system activity. This dynamic refinement effect finally during SPD leads to an inhomogeneously grain-refined two-phase microstructure, which may be responsible for the large hardness scatter observed. The inhomogeneity, respectively scatter of microhardness, becomes less with increasing shear strain and HPT pressure. During pressure release and/or temperature increase the hcp phase becomes unstable, and a reverse transformation is very likely. This process leads to a reduction of internal stresses and the formation of dislocation-free new grains of fcc phase. Consequently, as a result of this process, the microstructure and microhardness become quite inhomogeneous, too. However, the reverse transformation may be hampered by SPD-induced grain refinement.

### 4.3. Effect of Phase Transformation on Texture Formation

The texture of the fcc phase after HPT at RT and LNT has already been shown in [13,23,24]. It is a brass-type shear texture typical of fcc metals and alloys of medium/low SFE. The weak texture is even more randomized when martensite formation sets in. These results have been confirmed with statistically more secure texture measurements using high-energy synchrotron radiation. Reasons for the weak texture development discussed are twinning and grain refinement, the latter leading to possible grain boundary sliding [13,23]. Evidently, grain/interphase boundary sliding is more pronounced in the two-phase HEA. 

Using synchrotron radiation and a high-resolution 2D detector allows to analyze the texture of the hcp phase with high reliability, too. If the hcp phase is in the minority, its basal texture, according to the relationship given above, is determined by grains with fcc *A*-fibre orientations, see (111)_fcc_ and (002)_hcp_ PFs in Figure 8 and Figure 9. Such grains are preferentially oriented for shear on the {111}_fcc_ slip plane. If the hcp phase is the dominant phase, it develops a deformation texture of its own (*c*-axis fibre texture rotated anticlockwise about 30° towards SD) determined by the operative slip systems. As the *c/a* ratio of the hcp phase produced by a displacive transformation is ideal (*c/a* = 0.4142/0.2544 = 1.633 ≈ (*c/a*)_id_ = 1.633 [34]), basal and prismatic slip systems should have the same critical resolved shear stresses and therefore should contribute equally to deformation. It should be highlighted that for samples with minor and major contents of hcp phase, their hcp texture is relatively weak. It can be ascertained by the low mrd factor for (002)_hcp_ PFs of the hcp phase that amounts to about 7–8. For strong textures of hcp metals, it may reach up to 100. A texture simulation under such a condition is under way.

Another issue is the texture of the LNT HPT-deformed material during long-term storage at ambient conditions. As can be seen in Figure 12 a completely new texture component of the fcc phase emerges in the sample stored at RT and atmospheric pressure over two years. This 45° TD-rotated cube component indicates a recrystallization and/or grain growth process. Furthermore, two new components of the hcp phase appear. Interestingly, these two components are very good in line with the rotated cube orientation of the fcc phase. The orientation relationship can be easily recognized, when (111)_fcc_ and (002)_hcp_ PFs are compared in Figure 12. This strongly indicates that during the recrystallization and/or grain growth process not only is a new texture component of the fcc phase created, but new orientations of the hcp phase are also formed. Therefore, it is rather the nano grain size in combination with a high dislocation density that is unstable at RT, but not necessarily the hcp phase. Apparently, such a mechanism leads to the orientation relationship shown in Figure 1, which yields low-energy interphase boundaries. 

### 4.4. Effect of Phase Transformation on Strength Development

The enormous grain refinement of the Cantor alloy during RT and LNT HPT at 7.8 GPa leads to a nanocrystalline material with a steady-state crystallite size of about 24 nm [23]. The steady-state dislocation density is 3 × 10^16^ m^−2^ after HPT at RT. Surprisingly; it is a factor of two lower after HPT at LNT [13,14]. As observed for nanocrystalline Pd-10at.%Au, the transition from grain size softening to grain size hardening takes place at a grain size of approximately 20 nm [35]. From the maximum microhardness of 510 HV at RT and a pressure of 7.8 GPa a maximum stress σ_max_ ≈ 10 HV/3 = 1.7 GPa can be estimated, which represents about the maximum strength achievable at RT in the polycrystalline Cantor alloy. This value is about 1/20 of the theoretical strength σ_th_ ≈ *MG*/2π, with the shear modulus *G* = 79 GPa [36] and *M* = 3.07 the Taylor factor for a random orientation distribution. In the case of nanocrystalline Pd-10at.%Au a slightly higher value 1/15 was measured [35]. The strength determined here from microhardness compares well with that measured in tension (1.95 GPa [4]) and compression (2.0 GPa [37]). For the material deformed by HPT at LNT, the steady-state strength at RT (1.5 GPa) is approximately 15% lower. A strength anomaly (softening) was also observed by Scheriau et al. [16] for the austenitic steel A220. 

So far, the strength anomaly found for RT and LNT-HPT deformed materials with no or little hcp phase volume fraction based on X-ray line profile analysis has been discussed by dislocation nucleation and propagation in the parent fcc phase [13]. With a grain size of 24 nm, according to Hall–Petch [38,39], grain boundary strengthening via dislocation pile-ups are unlikely due to missing dislocation sources within the nano grains. Therefore, dislocation nucleation must occur through emission from grain boundaries. The stresses for nucleation of full (lattice) and partial dislocations (LD and PD) have been estimated to *σ*_LD_ = 2.6 GPa and *σ*_PD_ = 1.1 GPa, respectively [13]. Comparing these values with *σ*_max_ above, it has been concluded that nucleation of dislocations at the grain boundaries takes place in the form of partial dislocations. During the propagation of the dislocations through the nano grains, they experience lattice friction, interact with local fluctuations of solute concentrations [40], remnant dislocations, SFs, twin boundaries, and hcp lamellae. The Peierls stress (lattice friction stress at 0 K) of the fcc phase has been estimated by density functional theory calculations to be *σ*_F_ = *M* 0.178 GPa = 0.546 GPa [41]. Due to thermally activated kink formation, the friction stress is significantly smaller at LNT and RT [36]. The solid solution strengthening at 0 K is *σ*_SS_ ≈ 0.4 GPa [37]. Due to thermal activation, the stress contribution at higher temperatures is smaller, too [37]. The density of SFs and twin lamellae is low after HPT at RT due to nano grain size [13]. Consequently, their strengthening effect can be neglected. Hence, the stress for propagation of screw dislocations at RT is *σ*_*ρ*_ ≈ 1.7 GPa (Taylor hardening). For the material that is HPT-deformed at LNT, the interaction stress of screw dislocations changes to 1.2 GPa due to a lower dislocation density. In the case of edge dislocations, the hardening contribution increases to 1.6 GPa. Neglecting an additional contribution from the interaction of dislocations and hcp lamellae (including stacking faults) the latter value agrees well with 1.5 GPa measured in experiment [13]. Based on this agreement, it has been concluded that the yield stress of the nanocrystalline Cantor alloy with low volume fraction of hcp phase is predominantly determined by dislocation–dislocation interaction in the fcc phase. A similar conclusion was drawn by Podolskiy et al. [14] and Heczel et al. [42]. 

It is surprising that the dislocation density in the fcc phase after HPT at LNT is lower than that after HPT at RT. A plausible reason would be recovery during “self-annealing”, as was found for Cr_26_Mn_20_Fe_20_Co_20_Ni_14_ [43] in combination with a lowering of the microhardness. However, the latter effect has not been observed by Podolskiy et al. [14] for the Cantor alloy with time-dependent macro- and microhardness measurements at RT and LNT, i.e., without heating up to RT. They therefore hypothesized that the shear produced by the deformation-induced hcp phase formation leads to a reduction in strain of the fcc phase and hence a reduction in dislocation density. This argument is now invalidated by the results presented here for LNT HPT at different pressures showing that the microhardness does not decrease when the volume fraction of the hcp phase increases (Figure 16). 

It has to be noted that in the PtRu alloy (5 and 10 at.% Ru), the RT microhardness of the LNT HPT deformed sample is higher than that of the sample HPT-deformed at RT [44]. Furthermore, for pure fcc and bcc metals HPT deformed at LNT without recrystallization, the Leoben group observed stronger X-ray line broadening than for the RT-HPT deformed samples, indicating smaller crystallite size and/or higher dislocation density (unpublished). Thus, the softening behavior observed for the Cantor alloy and the austenitic steel A220 should be related to the martensitic phase transformation.

Based on the new results on microhardness and volume fraction of hcp phase changed by pressure, it is tempting to relate the hardness anomaly to the strength contrast between fcc and hcp phase. In a HEA of different composition (Cr_20_Mn_6_Fe_34_Co_34_Ni_6_) prone to the same phase transformation, compression of micro pillars shows that the hcp phase is harder than the fcc one [45]. This also explains the higher strength measured for the dual phase alloy Cr_10_Mn_30_Fe_50_Co_10_ [46]. Here, the strength of the hcp phase must be lower in order to account for the lower strength of the aggregate. Moreover, there should be a dependence on the volume fraction of the hcp phase, which is not observed. It should be emphasized that both phases during HPT experience severe plastic deformation, which may lead to different microstuctures and consequently to different strengths. X-ray profile analysis of both phases should bring to light the differences in microstructure with regard to crystallite size, dislocation density, dislocation character, and dislocation arrangement. Such an analysis of the high resolution diffractograms taken with synchrotron radiation is under way. 

Another reason for the hardness anomaly, may be the most important, seems to be the fact that during pressure release and/or temperature increase the hcp phase becomes unstable, and a reverse transformation is very likely. The reverse transformation can also be promoted by the stresses acting under the indenter. This process leads to a reduction of internal stresses and the formation of dislocation-free new grains of the fcc phase. Consequently, because of this process, the overall dislocation density of the fcc phase and the hardness of the polyphase aggregate are lowered. Moreover, the microstructure and microhardness become quite inhomogeneous, too. The decrease in microhardness of the specimens deformed at RT and 10 GPa and the large scatter of the data can also be explained in the same way.

With the HPT, a texture formation takes place, which can change the strength via the Taylor factor. However, due to twinning and grain refinement, the texture developing in the fcc phase during HPT is quite weak. It is a brass-type shear texture with dominant B resp. $\bar{B\;}$ and $A_{1}^{*}$ resp. $A_{2}^{*}$ and minor A resp. $\bar{A\;}$. It becomes almost random, when the phase transformation takes place. Based on this, it has been concluded that texture does not play a role in the strength anomaly [13]. The texture of the hcp phase is a weak basal texture if the hcp phase is low. It is related to the fcc texture according to the displacive transformation mechanism. There is a distinctive texture change to a deformation specific TD-rotated basal texture when the hcp phase becomes dominant. However, this does not affect the microhardness. Thus, the texture of both phases does not play a significant role in the strength anomaly.

Finally, it should be mentioned that RT microhardness measurements on austenitic steel (A220) after HPT at temperatures from 77 K up to 993 K have shown that the hardness almost linearly increases up to 803 K by 15% and then decreases due to recrystallization and/or grain growth [16]. Moreover, a phase transformation from fcc to hcp has been observed at LNT. As the steady state grain size slightly increases with increasing HPT temperature and as the dislocation density is expected to decrease, the observed hardness anomaly below 803 K is strange. However, assuming that the grain size is in the inverse Hall–Petch regime then the linear increase can be explained by grain/interphase boundary sliding [47]. Thus, the behavior below RT is similar to that observed for the Cantor alloy, but less intense. Therefore, the same strengthening mechanism may be assumed for both alloys. To prove the analogy at higher temperatures, similar experiments are planned for the Cantor alloy. It should be mentioned that, for grain/interphase boundary sliding dominated deformation, the grain size increase with temperature must be only 15%. Taking into account the fundamental problems of grain size measurements, this small increase cannot be checked reliably. The interfacial sliding process has the charm of describing both the softening and hardening phenomena below and above RT, respectively. From the texture evolution, it is concluded that interfacial sliding occurs in nanocrystalline materials even at low temperatures [13,23,35]. In contrast, Renk et al. [44,48], based on annealing experiments, concluded that the hardening above RT is best explained by the annihilation of lattice dislocations and grain boundary defects, complicating the absorption of dislocations at the grain boundaries after the heat treatments.

## 5. Conclusions

Based on investigations of microstructure, texture, and strength on the HPT-deformed prototype CrMnFeCoNi HEA, the following conclusions are drawn:

1. Under hydrostatic pressure CrMnFeCoNi HEA undergoes a phase transformation from fcc to hcp. The onset pressure of the displacive transformation is reduced by HPT, because shear stress promotes partial dislocation slip. The volume fraction of hcp phase increases with HPT pressure. Storage of the Cantor alloy HPT-deformed at LNT at ambient conditions leads to a reverse transformation. The time dependence observed complicates the study of the effect of martensitic transformation on the mechanical properties.
2. The texture of both phases depends on the phase fractions developing during the deformation-induced martensitic transformation. The texture of the fcc phase is a dominant brass-type shear texture. It is weak and randomizes with increasing fraction of hcp phase. The texture of the hcp phase is a weak basal texture for low fraction of hcp phase. It forms by the specific displacive transformation mechanism with an orientation relationship to the fcc phase. If the hcp phase is the dominant phase, the basal texture changes to TD-rotated, which may be regarded as a deformation texture of its own determined by the operative slip systems. The weakening of texture with shear strain indicates the contribution of grain/interphase boundary sliding in the nanocrystalline microstructure.
3. The microhardness of samples deformed at RT is above those deformed at LNT. This anomaly is related to the martensitic phase transformation. Assuming that during pressure release and/or temperature increase as well as during RT indentation the hcp phase becomes unstable, then a reverse transformation is very likely. This process leads to a reduction of internal stresses and the formation of dislocation-free new grains of fcc phase. Consequently, because of this process, the overall dislocation density of the fcc phase and the hardness of the polyphase aggregate are lowered. Moreover, the microstructure and microhardness become quite inhomogeneous, too. The decrease in microhardness of the specimens deformed at RT and 10 GPa and the large scatter of the data can also be explained in the same way.
4. The hardness anomaly of the HEA resembles that of austenitic steel. Assuming that the nano grain size falls into the inverse Hall–Petch regime, then the increase of RT hardness with increase of HPT temperature may be also controlled by grain/interphase boundary sliding. This assumption is reinforced by the fact that the amount of hcp phase affects hardness only slightly.

## Data Availability

The data presented in this study are available on request from the corresponding authors.
